# High‐intensity interval training improves acute plasma volume responses to exercise that is age dependent

**DOI:** 10.14814/phy2.13609

**Published:** 2018-02-21

**Authors:** Georges Jabbour, Horia‐Daniel Iancu, Hassane Zouhal, Pascale Mauriège, Denis R. Joanisse, Luc J. Martin

**Affiliations:** ^1^ Sport Science Program College of Arts and Sciences Qatar University Doha Qatar; ^2^ School of Kinesiology and Leisure Faculty of Health Sciences and Community Services Université de Moncton Moncton New Brunswick Canada; ^3^ Movement Sport and Health Sciences (M2S) UFR‐STAPS University of Rennes 2 Rennes France; ^4^ Centre de Recherche de l'Institut Universitaire de Cardiologie et de Pneumologie de Québec Québec Canada; ^5^ Département de kinésiologie Faculty of Medicine Université Laval Québec Canada; ^6^ Département de Biologie Université de Moncton Moncton New Brunswick Canada

**Keywords:** High‐intensity interval training, plasma volume, supramaximal cycling test

## Abstract

Plasma volume (PV) is affected by several factors including age, physical training and, acutely, by exercise intensity. The purpose of this study was to investigate the effects of 6 weeks of high‐intensity interval training (HIT) on PV and blood pressure (BP) changes among sedentary individuals. Thirty subjects aged between 18 and 71 years [body mass index=30.1(1.2) kg/m^2^] completed a 6‐weeks HIT program. Anthropometric and fitness variables were obtained at pre‐ and post‐ HIT. PV variations during warm‐up and after supramaximal cycling test (SCT) were calculated using two methods based on Hematocrit (Ht) and Hemoglobin (Hb) measures. After both the warm‐up and SCT, PV decreased significantly among participants at pre‐ and at post‐HIT (*P* < 0.01). However, PV decreases were significantly greater at pre‐HIT compared with post‐HIT during warm‐up and after SCT (*P* < 0.01, respectively). In addition, at pre‐HIT, a positive relationship was found between age and both PV variations at warm‐up and after SCT (*r* ^2^ = 0.55 and *r* ^2^ = 0.46; *P* < 0.01 respectively). However, no relationship was found during the post‐HIT period. After SCT and after both visits, only body weight predicted 22% of PV variations. In the current study, a significant relationship was found between systolic and diastolic BP improvements and PV variations in post‐HIT (*r* ^2^ = 0.54 and *r* ^2^=0.56, *P* < 0.05, respectively). Our results suggest that HIT may improve PV values and reduce the effects of age on the decrease in PV. These interventions led to improvements in systolic and diastolic BP values among participants.

## Introduction

PV variations are considered a form of body fluid adaptation in response to several factors such as age (Berthoin et al. 2003), training level (Moussa et al. 2003) and exercise intensity (El‐Sayed et al. 2011). Several investigators (Leaf 1984; Phillips et al. 1984) have observed that by aging individuals have difficulty maintaining body fluid balance, contributing to fluctuations in PV. This phenomenon has been observed at rest and acutely during exercise (Phillips et al. 1993), with larger PV decreases observed in response to exercise (Hebestreit et al. 1996). These PV decreases affect heart rate and impact blood flow to active muscles (Kenney and Ho 1995).

It is well known that exercise training, particularly the endurance exercises (cycling or running) (Krip et al. 1997; Zouhal et al. 2009) result in significant increase in PV, leading to an increase in exercise performance (Coyle et al. 1990; Luetkemeier and Thomas 1994). In older individuals (~55 years), Hagberg et al. (1998) showed that those who were aerobically trained had greater PV than their sedentary counterparts, leading to higher cardiac stroke volume and cardiac output at a given relative intensity of exercise. However, several studies show that the increase in PV following aerobic training was lower in older than in younger subjects (Stachenfeld et al. 1998; Takamata et al. 1999), and this might result from reduced fluid intake after thermal dehydration (Takamata et al. 1999) or water deprivation (Phillips et al. 1984) in these older individuals. To date, data on PV variations in response to training in older adults are limited to resting measures.

On the other hand, several studies have shown that a short‐training period involving intermittent exercises may result in significant variations in PV (Green et al. 1987; Gibala et al. 2012). Indeed, Green et al. (1987) observed in moderately trained subjects an increase of 11.6% of PV in response to only three consecutive days of intermittent supramaximal interval cycling exercise. High‐intensity interval training (HIT) is a type of exercise that combines short high‐intensity exercise periods with rest or low‐intensity exercise periods (Gibala et al. 2012). Early studies have shown the effectiveness of this exercise model in improving several health and performance indicators, even after short intervention periods (Whyte et al. 2010; Gillen and Gibala 2013). While most evidence is based on relatively younger adults, other studies have reported similar results in elderly (Hwang et al. 2016). Although exercise intensity is an important factor governing the hemoconcentration effect as well as PV during interval exercise (Bloomer and Farney 2013), little is known, however, about the effects of HIT on PV variations and its eventual link to the improvement of fitness and/or of health parameters.

Finally, it is common that hypertension rises with age (Burt et al. 1995), and BP control represents a relevant clinical target. Studies investigating the impact of exercise training on hemodynamic disturbances among elderly individuals reported significantly decreases in resting BP after prolonged endurance training (Kelley et al. 2001) and after strength training (Kelley and Kelley 2000). Because of the dependence of BP on PV (Julius et al. 1971), any variations in PV might impact BP values. To the best of our knowledge, no data have addressed the potential role of PV variations on BP improvement in response to training. According to data in trained adults, HIT may result in significant variations in PV. Therefore, evaluating the potential benefits derived from HIT on PV and its eventual link to BP adaptations might provide additional support for recommending HIT among sedentary individuals.

Hence, this study examined the effects of HIT on PV variations among sedentary wide age range subjects and its relationship to BP. For this purpose, we measured PV before and after 6 weeks of HIT, as well as acute variations to PV after warm‐up and at the end of supramaximal cycling exercise. We hypothesized that a short HIT program would increase PV and reduce the age‐related effect on PV decreases in response to exercise. Furthermore, we hypothesized that this adaptation would contribute to the improving BP in these participants following training.

## Materials and Methods

Thirty voluntarily healthy individuals (10 men and 20 women) who met the inclusion criteria were recruited from the University of Moncton staff after posting announcements throughout the University campus. Our initial sample was 35 participants, however, two subjects did not meet eligibility requirements, and three declined to participate. Mean age was 38 years (range, 18–71 years). The study protocol was approved by the University's Human Research Ethics Committee (UHRC) and all procedures were followed in accordance with the Helsinki Declaration of 1975 as revised in 2008. Informed consent was obtained from all subjects prior to being included in the study. The inclusion criteria for participation were as follows: participants had to be sedentary, participating in <60 min/week of structured exercise, as assessed by the International Physical Activity Questionnaire (Craig et al. 2003), and none of them participated in any systematic exercise training at the time of study enrollment or during the 6 months preceding the experiment; moreover, they had no history of orthopedic, neurological, cardiovascular or other chronic disease, no history of drug consumption before the study, and no history of smoking. Throughout the intervention and prior to measurements, participants were asked not to consume alcohol and were encouraged to eat their normal diet and maintain their typically sedentary behavior so as to not affect the outcome variables. In addition, participants were instructed not to actively commute (walk and or cycle) during the study. Before entering the protocol, each participant was thoroughly familiarized with all testing equipment and procedures.

The protocol then began with preliminary testing to determine baseline levels of key variables (pre‐HIT testing). The testing was conducted on three different days (day one: D1, day two: D2 and day three: D3) after an overnight fast, and took place in the morning of each day (~8 h 30). The three testing days were separated by a minimum of 48 h, and all subjects were asked to avoid physical activity for 48 h prior to each testing session. All participants completed all training sessions and no other difficulties were encountered.

### Anthropometric measurements

Body mass, body fat percentage, fat‐free mass and fat mass were assessed using bioelectrical impedancemeter (Vacumed Bodystat1500, Isle of Man, British Isles). Height was determined to the nearest 0.5 cm with a measuring tape fixed to the wall. Body mass index (BMI) was calculated as the ratio of body mass (kg) to height^2^ (m^2^).

### Physiological assessments

At baseline on D1, participants rested in a supine position for at least 30 min before BP measurements that were taken using an automated BP monitor (Omron HEM705 CP; Omron Healthcare, Milton Keynes, UK). On each occasion, 3 measurements of BP were taken and the lowest values were used for analysis. Next, participants performed a maximal test on an upright cycle ergometer (Monarkergomedic 839E electronic test cycle, USA) to determine their maximal oxygen consumption (VO_2_max). The participants cycled at an initial power of 25 W, which was then progressively increased by 25 W every 2 min until exhaustion. A breath‐by‐breath automated metabolic system (Ergocard MEDI‐SOFT, Dinant, Belgium) was used to determine gas exchange. Maximal oxygen consumption was deemed to have been reached when a subject fulfilled at least three of the following criteria: a plateau in VO_2_ despite an increase in exercise intensity; a respiratory exchange ratio greater than 1.1; a maximal HR above 90% of the predicted maximal theoretical heart rate (220 – age in years) or apparent exhaustion (Spiro 1977). All participants met this requirement. During this maximal test, heart rate values were continuously measured using an electrocardiogram (CASE 16 exercise testing system, Marquette, Wisconsin, USA).

On D2, following 10 min of warm‐up, the participants performed a Charge‐Velocity (Ch‐V) test on a cycle ergometer using a technique adapted from the study of Vandewalle et al. (1988). This test consisted of a succession of supramaximal bouts of approximately 6 sec, with flywheel resistance increasing by 1 kg after each bout until the subject was unable to perform the test. A period of passive recovery (5 min) was allowed between successive bouts. Peak velocity for each bout was recorded, and power output was calculated by multiplying the load with speed. The optimal load corresponded to the load at which maximal power (*P*_max_) was achieved. This load was then used for the training protocol that followed. The charge‐velocity test was also performed every 2 weeks to adjust individual power levels of supramaximal cycling test (SCT). These 2 days of testing took place before (pre‐HIT) and again at the end of the training period (post‐HIT) following the same procedures.

On D3, each subject performed a repeated SCT (6 repetitions of 6‐second maximal sprints with 2 min of passive rest between each repetition). The experiment began for all subjects at approximately 8 h 30 am. Venous blood samples from an antecubital vein were drawn after 5 min of rest in an upright position on the cycle ergometer, and then participants warmed up for 5 min at a submaximal power corresponding to 60% of VO_2max_. Venous blood samples were again drawn after the warm‐up period and immediately after the end of the SCT.

At each extraction by venipuncture, blood was collected in a vacutainer tube containing Ethylene Diamine Tetra Acetic Acid (EDTA*)*. Hematocrit (Ht) was measured three times for each blood sample by microcentrifugation (JOUAN‐HEMAC). Plasma from the venous blood samples was separated by centrifugation at 3000*g* for 20 min (4°C) (ORTO ALRESA mod. Digicen R, Spain). Aliquots were immediately frozen and stored at −80°C for use in subsequent chemical analyses. PV variations, expressed as percentages of resting values, were calculated from Ht variations according to Van Beaumont (1972): %PV variations = 100 [(Ht1 − Ht_*x*_)/Ht_*x*_ × (100 − Ht1)], where Ht1 is the resting value and Ht_*x*_ is the value at the time of exercise, after the warm‐up period or at the end of SCT. In this study, we used also the method of Dill and Costill (1974) based on measurements of the Ht and the hemoglobin concentration (Hb) using the equation: %PV variations =  100 × [(Hb1/Hb_*x*_) × (100 − Ht_*x*_)/(100 −Ht1)] − 1.

### Training sessions

Once participants completed preliminary testing, they were instructed to complete 18 training sessions (three sessions per week for 6 weeks), following the protocol developed by our laboratory (Jabbour et al. 2017). Each session began with a 5‐min warm‐up of continuous cycling at moderate intensity corresponding to 40–50% of their HRmax followed by 6 repetitions of SCT intervals with 2 min of passive recovery between each repetition. Each SCT repetition lasted 6 sec, and participants were asked to pedal at maximal velocity against the resistance determined during the Ch‐V test. The total duration of each session was approximately 15 min.

Training sessions were conducted under the supervision of a member of the research team, and velocities (in RPM) were recorded for each second of the session to ensure that they remained constant. Post‐HIT anthropometric, fitness, PV, and BP measurements (similar as D1, D2 and D3) were conducted after the final training session to obtain information on the potential chronic lasting effects of HIT.

### Statistical analysis

Data are presented as means (standard error). Normality was tested using the Kolmogorov–Smirnov test. Paired *t*‐tests were used to determine whether significant changes occurred in our primary variables (i.e., fitness, anthropometry, BP and PV) between pre‐ and post‐HIT. The relationship between variations in PV, BP and age between visits were assessed via Pearson correlations. Multiple linear regression with an extended‐model approach was subsequently used to document the effects of the variables on PV variations. As a result, a series of multiple linear regression models were developed to determine the relationship between each variable and PV variations. A value of *P* < 0.05 was considered statistically significant. Analyses were performed using IBM SPSS Statistics 19 software (IBM SPSS Statistics for Windows, Version 19.0. Armonk, NY: IBM Corp.).

## Results

Subject characteristics are presented in Table 1. Following 6 weeks of HIT, neither anthropometric variables nor VO_2_max changed compared to pre‐HIT. However, diastolic BP (78.3 vs. 71.6 mmHg) and systolic BP (114.5 vs. 109 mmHg) decreased significantly post‐HIT (both *P* < 0.001) compared to pre‐HIT.

**Table 1:** Age, anthropometric, physiological and fitness variables of participants

	Pre‐HIT	Post‐HIT	Paired *t*‐test	
			*T*	*P*
Age (years)	38 (30)	–		
Height (cm)	169 (30)	–		
Weight (kg)	83.2 (3.1)	84.1 (3.1)	1.5	0.13
FFM (kg)	56.3 (2.1)	57.3 (2.6)	1.7	0.18
FM (kg)	30.1 (2.5)	29.6 (1.5)	2.3	0.21
BMI (kg/m^2^)	30.1 (1.2)	29.9 (1.2)	1.3	0.11
Systolic blood pressure (mmHg)	114.5 (2)	109.1 (2.2)	39.8	<0.001
Diastolic blood pressure (mmHg)	78.3 (1.6)	71.6 (1.6)	23.8	<0.001
HR rest (beats/min)	74 (2)	76 (2)	1.8	0.21
VO_2max_ (mL/min/kg)	26.9 (1.4)	27.2 (1.2)	1.3	0.11
HR_max_ (beats/min)	180 (2)	178 (2)	1.6	0.17
RER	1.1 (0.05)	1.1 (0.03)	2.8	0.31

Values are given as the mean (standard error). FFM, fat free mass, FM, fat mass, BMI, body mass index, VO_2max_, maximal oxygen consumption, HR, heart rate, RER, respiratory exchange ratio.

During exercise, hematocrit increased significantly from rest to warm‐up (*P* = 0.14) and again after SCT (*P* = 0.26) at pre‐ as well as at post‐HIT. These variations were significantly greater at pre‐ compared to post‐HIT (*P* < 0.01) (Table 2).

**Table 2:** Mean values of hematocrit, hemoglobin and PV variations

	Pre‐HIT	Post‐HIT	Paired *t*‐test	
			*T*	*P*
Rest				
Hematocrit (%)	40.4 (3.1)	39.9 (2.1)	1.4	0.18
Hemoglobin, g (100 mL)^−1^	16.4 (0.3)	16.1 (0.3)	1.1	0.12
After warm‐up (Van Beaumont method)				
Hematocrit (%)	43.9 (4.6)^2^	41.1 (3.3) 2	1.1	0.14
PV variations (%)	−13.3 (0.6) 2	−4.8 (1.3) 1 ^,^ 2	22.1	<0.001
After warm‐up (Dill and Costill method)				
Hemoglobin, g (100 mL)^−1^	16.4 (0.1)	16.1 (0.4)	1.3	0.22
PV variations (%)	−12.9 (0.3) 2	−4.3 (1.3) 1 ^,^ 2	17.1	<0.001
End of SCT (Van Beaumont method)				
Hematocrit (%)	46.5 (1.2)^1^^,^ 2	44.1 (3.3) 1, 2	1.3	0.26
PV variations (%)	−22.2 (0.7)^1^^,^ 2	−15.5 (0.4)^1^^,^ 2	31.1	<0.001
End of SCT (Dill and Costill method)				
Hemoglobin, g*·*(100 mL)^−1^	16.1 (0.3)	16.1 (0.2)	1.1	0.12
PV variations (%)	−23.3 (0.4) 2	−14.9 (0.3) 1 ^,^ 2	16.1	<0.001

Values are given as the mean (standard error). PV, plasma volume. Significant difference from warm up (^1^ *P* < 0.01). Significant difference from rest (^2^ *P* < 0.01).

**Figure 1:**
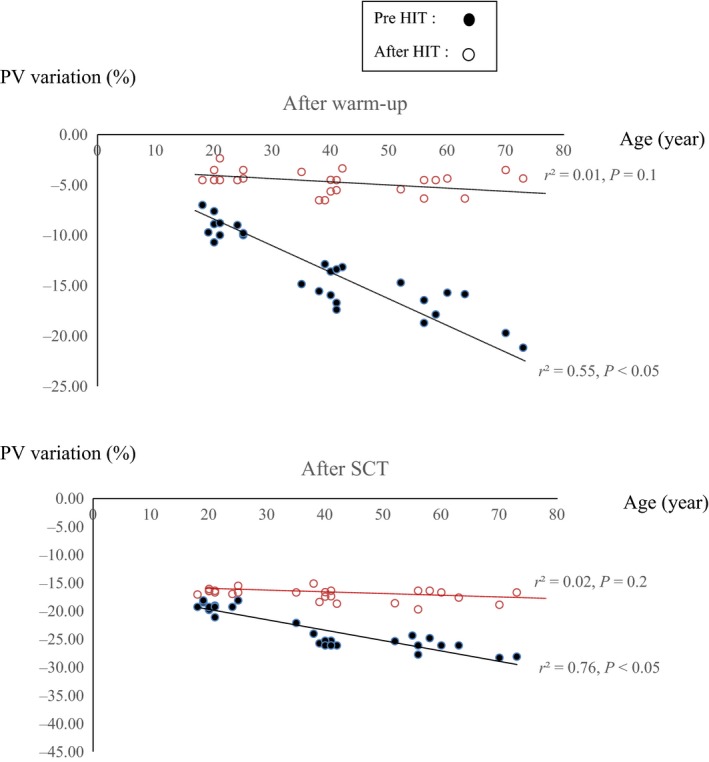
Relationships between age and PV variations at pre‐ and post‐HIT.

At baseline, a positive and significant correlation was found between PV decreases and age among participants during both warm‐up (*r* ^2^ = 0.55, *P *< 0.05) and at the end of SCT (*r* ^2^ = 0.46, *P* < 0.05) (Fig. 1). However, no significant correlation was found between these variables at post‐HIT (*r* ^2^ = 0.01; *P* = 0.1 and *r* ^2^ = 0.02; *P* = 0.2).

Multiple linear regression analysis demonstrated that age contributed significantly to PV decreases before training after warm‐up (*r* = 0.66) and after SCT (*r* = 0.76). Following HIT, no age effect on PV decreases was detected for warm‐up and for SCT. In this study, a significant correlation was observed between systolic and diastolic BP improvements and PV increases determined after warm‐up (*r* ^2^ = 0.53 and *r* ^2^ = 0.58, *P* < 0.05, respectively) and at SCT (*r* ^2^ = 0.54 and *r* ^2^ = 0.56, *P* < 0.05 respectively).

## Discussion

To the best of our knowledge, this study is the first to examine PV variations in response to HIT in subjects from a broad age group (18–71 years). Our results show that acute exercise‐induced PV decreases were greater in participants before training, and age contributed significantly to explaining these PV variations and this for both methods of Van Beaumont (1972) and Dill and Costill (1974). However, following HIT, acute PV decreases were attenuated compared to those observed at pre‐HIT, and the relationship with age was lost.

To date, there is no information on PV response to exercise among older individuals. However, according to data provided by Mtinangi and Hainsworth (1999), significant increases in PV were reported after 2–3 months of exercise training using the 5BX/XBX Royal Canadian Air Force programme among 11 volunteers (range 23–68 years). Furthermore, Alis et al. (2015) reported higher PV loss after an acute HIT protocol in 10 healthy men (~25 years) compared to an aerobic running exercise. Nevertheless, there are no data regarding PV variations in response to such training models.

It has been shown that acute HIT results in a sufficient decrease in PV to increase plasma constituent concentrations (Alis et al. 2015). In this study, prior to training, submaximal (warm‐up, 5 min at a power corresponding to 40–50% of HRmax) or SCT exercise resulted in decreased PV that was positively and significantly associated with age. Accordingly, Hebestreit et al. (1996) reported 20% decreases in PV in 5 men aged 19–29 years, performing intense exercise. The aging process impairs balance of fluids and electrolytes (Rolls and Phillips 1990), leading to reduced PV among elderly individuals (Phillips et al. 1993). Considering that exercise intensity affects PV variations, we can speculate that this factor combined with advancing age potentiates PV decreases in older individuals.

Following HIT, our results showed less change in PV in response to warm‐up and SCT. To the best of our knowledge, no other study has evaluated acute PV variations in response to exercise following HIT in younger and older sedentary individuals. Most studies showing PV increases have been conducted in fit populations (i.e., athletes, active subjects). Studies that have evaluated the training effects on PV variations reported a significant increase in PV in response to exercise. For Abderraouf et al. (2013), 7 weeks of intermittent endurance training led to increased PV in active adult men (20 to 23 years old), and similar results have been reported in response to long‐term or relatively short‐term continuous exercise training (Green et al. 1990; Convertino 1991; Hodges et al. 2010). After a short training period, involving intermittent exercises (especially cycling), Green et al. (1987) observed an increase of 11.6% in PV in moderately trained subjects. Actually, no data exist until now to explain previous results (i.e., PV increases after training). However, if we assume that PV decreases depend mainly on capillary hydrostatic pressure (Selby and Eichner 1994) on body fluids, the response of the hormones controlling the hydro‐electrolytic equilibrium (Freund et al. 1987) and solute concentrations, it can be speculated that any improvement in one or some of these mechanisms could lead to PV improvements. However, this assumption cannot be fully considered, given that PV response can be affected by postural changes (El‐Sayed et al. 2011), hydration (Van Beaumont 1972), and time of day (Ahmadizad and Bassami 2010). Therefore, it is difficult to compare the results of studies using different training models in terms of duration and intensity, for example, and laboratory methods.

Interestingly, the relationship between age and acute PV decreases during exercise was lost following HIT in our participants. Actually, the aging process is characterized by changes in the regulation of homeostasis, potentially leading to impaired fluid and electrolyte balance (Alis et al. 2015). As reported by Kenney and Ho (1995), Kenny et al. (1997) and Phillips et al. (1993), the elderly are at risk of dehydration due to a decrease in total body fluids, blood viscosity and blood flow. Mora‐Rodríguez et al. (2015) showed that increased physical activity is associated with improved hydration status (i.e., lower urine and blood osmolality) among healthy men and women (20–60 years). For these authors, the PV improvement could be explained by an increase in hydration status and water turnover, which is probably a form of exercise training adaptation that raises body water content.

Considering that hemodynamic disturbances are frequent in the elderly, it is important to know the potential benefits derived from HIT on BP. Recent data have shown decreases in resting BP after prolonged endurance training (Kelley et al. 2001) and after strength training (Kelley and Kelley 2000). These positive effects caused by physical training were more pronounced in hypertensive participants (Cornelissen and Fagard 2005). Participants in this study had normal DBP (<80 mmHg) and SBP (<140–159 mmHg) at pre‐HIT. Interestingly, after training, both DBP and SBP were reduced (~5 and ~6 mmHg), which may have clinical and biological relevance in reducing the risk of heart disease. Because of the dependence of BP on PV, when PV decreased, and total peripheral resistance increased (Julius et al. 1971). Therefore, any variations in PV could impact blood pressure values. In fact, our results showed a significant relationship between systolic and diastolic BP improvements and PV variations after warm‐up and SCT following HIT.

In the present work, some limitations should be considered. In fact, the dehydration indexes (urine and blood osmolality) were not assessed; as a result, further data are needed to explore the effect of this phenomenon on PV variations in response to HIT among older individuals. Moreover, studying the effect of HIT on PV variations based on age categorization will be necessary to clarify how age in different categories (i.e., middle age, advanced age, etc.) may affect our variables.

## Conclusion

In conclusion, HIT might be recommended as a strategy aimed at reducing the age effect on PV decreases and improve PV values. Our analyses reveal that PV improvement observed among participants lead possibly to improve systolic and diastolic BP. An additional result from this study is that at baseline the age contributed significantly to PV decreases before training after warm‐up and after SCT. Therefore, HIT protocols should be carefully considered for older individuals with hypertension or other hemodynamic disorders, given that such interventions can increase losses. This latter may constitute a potential risk factor, since it could increase whole blood viscosity, vascular resistance, and the risk of suffering from a cardiovascular event.

## Conflict of Interest

The authors declared no conflict of interests regarding the publication of this manuscript.
